# The role of hippocampal subfield and medial temporal lobe volumes in subjective cognitive decline

**DOI:** 10.1002/alz70861_107966

**Published:** 2025-12-23

**Authors:** Ishitaa Arikirevula, Susan Vandermorris, Nicolaas Paul L.G. Verhoeff, Negar Mazloum‐Farzaghi, Rosanna K Olsen, Nathan Herrmann, Linda Mah

**Affiliations:** ^1^ Baycrest Health Sciences, Toronto, ON Canada; ^2^ Baycrest Health Science, Toronto, ON Canada; ^3^ Psychiatry, University of Toronto, Toronto, ON Canada; ^4^ University of Toronto, Toronto, ON Canada; ^5^ Toronto Dementia Research Alliance, Toronto, ON Canada; ^6^ Department of Psychiatry, Temerty Faculty of Medicine, University of Toronto, Toronto, ON Canada; ^7^ Baycrest Hospital, Toronto, ON Canada; ^8^ Rotman Research Institute, Baycrest Health Science Centre, Toronto, ON Canada; ^9^ Rotman Research Institute, North York, ON Canada; ^10^ Rotman Research Institute, Toronto, ON Canada; ^11^ Temerty Faculty of Medicine, University of Toronto, Toronto, ON Canada; ^12^ Sunnybrook Health Sciences Centre, Toronto, ON Canada; ^13^ Hurvitz Brain Sciences Program, Sunnybrook Research Institute, Toronto, ON Canada; ^14^ Neuropsychopharmacology Research Group, Sunnybrook Research Institute, Toronto, ON Canada; ^15^ Sunnybrook Research Institute, Toronto, ON Canada; ^16^ Centre for Addiction and Mental Health, University of Toronto, Toronto, ON Canada; ^17^ Baycrest Academy, Toronto, ON Canada

## Abstract

**Background:**

Subjective cognitive decline (SCD), defined as self‐perceived memory decline with normal test performance, may indicate preclinical Alzheimer’s disease (AD). While medial temporal lobe (MTL) atrophy is a key neuroimaging biomarker for AD, specific atrophy patterns in SCD remain unclear. Neuroimaging studies also indicate that hippocampal subfields, particularly the CA1 and subiculum, are among the earliest affected in AD. However, the literature on volumetric alterations in the hippocampal subfields and other MTL subregions in SCD is inconsistent. One potential reason is failure to account for previous diagnosis of depression, which itself is associated with hippocampal atrophy, in SCD samples. This study compared MTL and hippocampal subfield volumes in older adults with SCD and cognitively unimpaired (CU) adults without prior or current depression and examined their associations with quantitative measures of subjective memory.

**Method:**

Fifty‐one participants (26 SCD (13M, age 70.6 ± 5.1) and 25 CU (10M, age 71.4 ± 7.4) without psychiatric history completed the Memory Functioning Questionnaire (MFQ) and high‐resolution magnetic resonance imaging (MRI) of the MTL at 3Tesla. The Automatic Segmentation of Hippocampal Subfields (ASHS) pipeline and manual segmentation were used to extract regions‐of‐interest (ROIs). ANCOVA was used to compare group differences in ROIs. Regression analyses were conducted to examine associations between MFQ scores and ROIs.

**Result:**

Relative to CU, parahippocampal volume was smaller bilaterally in SCD [left: F(1, 48 =5.87, *p* =0.019; right: F (1, 48)=5.35, *p* =0.026], after controlling for age, sex, and total intracranial volume. A trend towards smaller left subicular volume was observed in SCD [F(1,48)=3.86, *p* =0.055]. Multivariable regression analyses controlling for age, sex, and intracranial volume showed positive associations between left subiculum volumes and MFQ‐Seriousness of Forgetting subscale scores, and between left CA1 volumes and MFQ‐Frequency of Forgetting subscale scores.

**Conclusion:**

These preliminary findings implicate the subiculum and parahippocampal gyrus in the neurobiology of SCD and suggest a predominant effect of the left hemisphere. Further, these data are not confounded by the presence of past or current depression in our sample. These findings require replication using larger samples of SCD with biomarker evidence of AD neuropathology.

